# Accidental intra-arterial injection of enoxaparin sodium leading to abdominal wall expanding subcutaneous hematoma and abdominal wound: case report-vascular

**DOI:** 10.3389/fsurg.2025.1477926

**Published:** 2025-01-22

**Authors:** Caroline Howell, Richard Simman

**Affiliations:** ^1^ University of Toledo College of Medicine and Life Sciences, Toledo, OH, United States; ^2^ Division of Plastic and Reconstructive Surgery, Department of Surgery, University of Toledo Medical Center, Toledo, OH, United States; ^3^ ProMedica Health Network, Wound Care Program, Jobst Vascular Institute, Toledo, OH, United States

**Keywords:** Lovenox, subcutaneous hematoma, anticoagulant, negative pressure wound therapy, bleeding, intra-arterial injection

## Abstract

**Introduction:**

Enoxaparin sodium (Lovenox®) is a commonly used anticoagulant medication that is self-administered via subcutaneous injection to prevent the formation of pathologic blood clots. It is used as a bridge to long-term anticoagulation with warfarin in patients at high risk for thromboembolic events. It is generally well-tolerated and has a favorable safety profile. The most common injection site reactions caused by enoxaparin sodium are urticaria, ecchymosis, and skin and fat necrosis.

**Case Report:**

A 56 year-old female with extensive thromboembolic history was completing an enoxaparin sodium bridge to warfarin when she accidentally self-injected enoxaparin sodium into the left superficial epigastric artery, resulting in the formation of a large expanding hematoma and the development of hemorrhagic shock. Controlling the bleeding required reversal of anticoagulation, transfusion, and coil embolization of the affected arteries. Surgical evacuation of the hematoma was performed, and the resultant wound was managed postoperatively with negative pressure wound therapy (NPWT) for one month. After discontinuation of NPWT, the wound was allowed to heal by secondary intention using dressing changes.

**Conclusions:**

The findings of this case report suggest that NPWT followed by conventional dressings can be used to close and heal the wound created by surgical hematoma evacuation.

## Introduction

Enoxaparin sodium (Lovenox®) is low molecular weight heparin used to treat and prevent thrombosis and embolism. It works by increasing the activity of antithrombin III, thereby inactivating factor Xa and shutting down the common pathway of the coagulation cascade (1, 2). It also inhibits factor IIa (thrombin), however, it has less anti-factor IIa activity than unfractionated heparin (1, 2).

One of the uses of enoxaparin sodium is as a bridge to long-term anticoagulation with warfarin for patients at high risk of thrombosis (3, 4). Enoxaparin sodium is useful as a bridging agent because it has a rapid onset of action, so concurrent initiation with warfarin ensures anticoagulation during the window of time before warfarin takes effect. Enoxaparin sodium can be self-administered by the patient subcutaneously using a preloaded syringe with 27-gauge, half-inch needle (5). Enoxaparin sodium is injected into either the left or right side of the abdomen, at least 2 inches lateral to the umbilicus (5). The short needle length, shallow injection depth, and injection site with few underlying superficial arteries makes accidental intra-arterial injection of enoxaparin sodium unlikely.

The most common adverse reactions of enoxaparin sodium are bleeding, anemia, thrombocytopenia, elevation of serum aminotransferase, diarrhea, and nausea (5). In addition, skin changes at the injection site such as urticaria, ecchymosis, skin and fat necrosis have been reported, as well as abdominal wall hematoma (6–8). In this paper, a case of accidental intra-arterial injection of enoxaparin sodium into the left superficial epigastric artery is described. The incident resulted in the formation of an expanding hematoma and development of hemorrhagic shock. This appears to be the first case of its kind reported. The case and its management are detailed below.

## Case description

A 56 year-old female with a complex medical history including bilateral carotid artery stenosis, stroke, multiple transient ischemic attacks, diastolic congestive heart failure, chronic obstructive pulmonary disease, type 2 diabetes mellitus, chronic pancytopenia, and liver cirrhosis with portal hypertension and esophageal varices due to nonalcoholic steatohepatitis was referred to the wound center for wound management. The patient was recently hospitalized for decompensated liver cirrhosis with portal and splenic vein thrombosis while on anticoagulation. During admission, the patient's home warfarin was reversed and held, and anticoagulation with heparin was initiated. She was discharged on three anticoagulants due to high thromboembolic risk: clopidogrel 75 mg daily, warfarin 7.5 mg daily, and enoxaparin sodium subcutaneous injection 100 mg/ml every 12 h for 14 days as a bridge to warfarin. Prothrombin time (PT) and international normalized ratio (INR) at time of discharge were 12.3 s and 1.1, respectively. Six days after discharge, the patient presented to the emergency department with abdominal pain following enoxaparin sodium injection and was found to have an expanding abdominal wall hematoma. Preliminary tests revealed a prothrombin time (PT) of 24.6 s and international normalized ratio (INR) of 2.4. Activated partial thromboplastin time (aPTT) was not reportable. Thrombin time and anti-Xa levels were not measured as this was not part of the hospital's standard protocol and clinicians did not feel these labs would change management given that the cause of the bleed could be reasonably inferred from the patient's history of chronic anticoagulation, elevated INR, and the temporal relationship between the enoxaparin sodium injection and development of the abdominal hematoma. Anticoagulation reversal was initiated; the patient was given 2 mg of intravenous (IV) Vitamin K and 1 unit of fresh frozen plasma (FFP). The patient was not felt to be a good candidate for protamine sulfate given the severity of her systemic disease including cardiac and respiratory conditions, insulin-controlled diabetes, and thrombotic history. There was concern that administration of protamine sulfate could lead to overcorrection and worsen her underlying coagulopathy. Additionally, the patient's labs (PT and INR) did not suggest severe or emergent anticoagulation. The hematoma was noted to increase in size by 2 cm while patient was in the emergency department.

**Figure 1:**
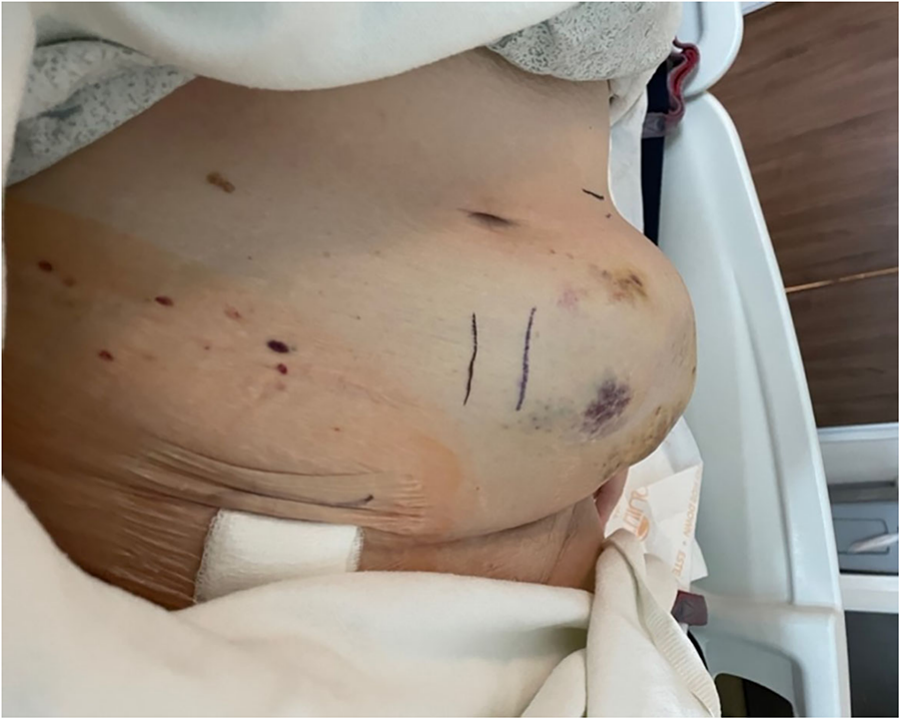
Abdominal wall expanding hematoma at the enoxaparin sodium injection site following accidental intra-arterial injection into the left superficial epigastric artery.

The patient was transferred to a larger hospital for care. After transfer, laboratory tests revealed PT (20.2 s), aPTT (66 s), INR (1.8), hemoglobin (6.7 g/dl), platelets (99,000/*μ*l). Blood pressure was 117/57 mmHg. The patient was given an additional 2.5 mg of Vitamin K, 1 unit of FFP, 1 unit of platelets, and 2 units of packed red blood cells (PRBC) and admitted to the medical intensive care unit (ICU). Upon admission, the hematoma measured 30 cm × 10 cm (Figure 1). Anticoagulants and antiplatelets were held. On hospital day 2, the abdominal wall hematoma was still bleeding. The patient developed hypovolemic hemorrhagic shock (lowest blood pressure 76/45 mmHg) and required blood transfusion and IV norepinephrine for 4 h. Clinically, the patient's abdomen remained distended and painful. Observation and serial hematoma measurements were deferred in favor of rapid intervention to stop the bleeding.

## Diagnostic assessment

**Figure 2:**
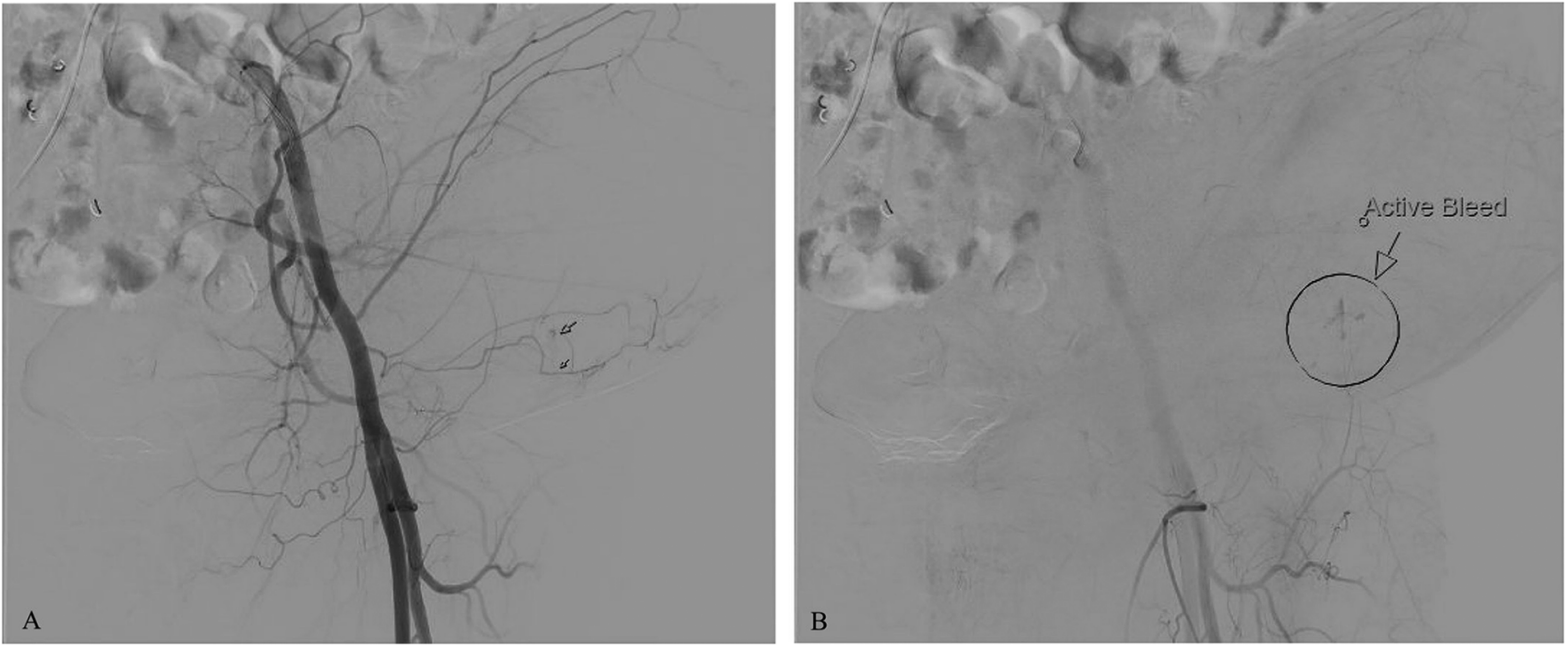
Arteriograms showing contrast extravasation from the left superficial epigastric artery. **(A)** Contrast extravasation observed during the initial injection; **(B)** Area of active bleeding indicated by an arrow and circle.

**Figure 3:**
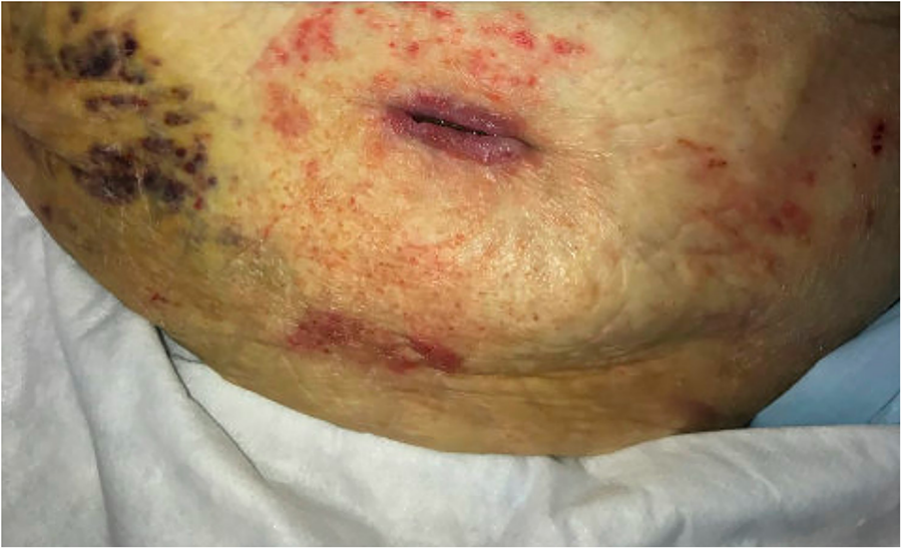
Healing left abdominal wall surgical wound upon presentation to wound center 26 days after discharge. Wound measured 2.2 cm long and 2 cm deep. NPWT was discontinued this day.

**Figure 4:**
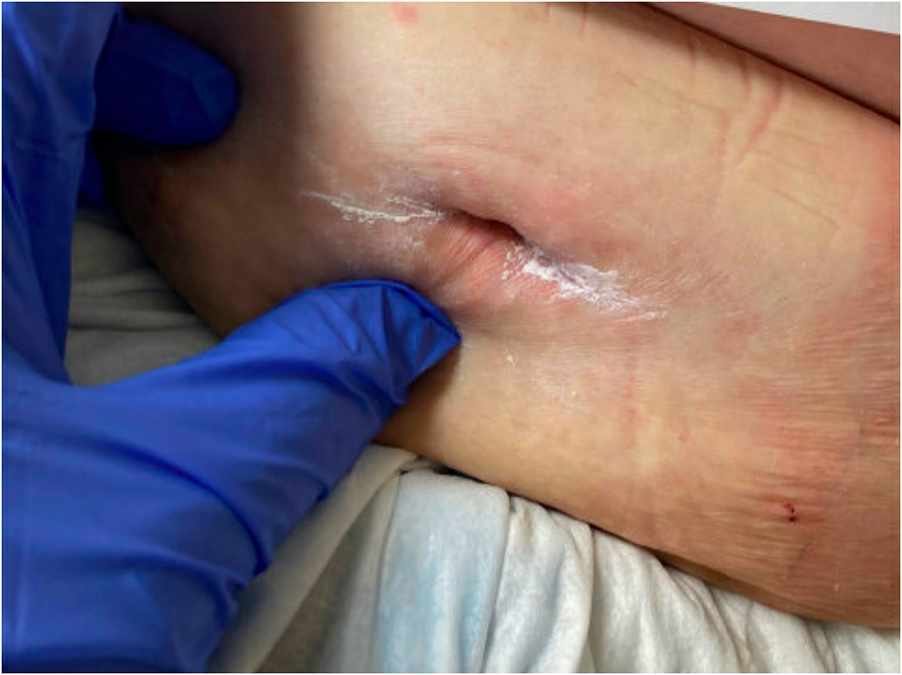
Wound appearance 54 days after discharge. Wound measured 1.2 cm long and 0.5 cm deep. Wound packing was discontinued this day.

To identify the source of bleeding, interventional radiology performed ultrasound-guided angiography. Access was obtained via the right common femoral artery. Arteriograms were performed for the right and left common femoral, left superficial epigastric, and left circumflex iliac arteries. Contrast extravasation was noted from the left superficial epigastric artery, as seen on arteriograms in Figures 2A,B. To control the bleeding, coil embolization of the left superficial epigastric artery was performed, in addition to empiric embolization of the left circumflex artery. On hospital day 3, surgical evacuation of the hematoma was performed, draining over 500 ml of blood. A negative pressure wound therapy (NPWT) device was applied in the operating room under general anesthesia. NPWT was applied continuously at −125 mmHg with instruction to decrease pressure to −100 mmHg if there were signs of continued wound bleeding or serosanguineous drainage. The foam covered approximately 50 square centimeters. Because the patient was high risk for bleeding, a non-adhering wound dressing (Adaptic®) was placed between the wound bed and the foam to minimize adherence. Dressings were changed three times per week. On hospital day 4, the patient was transferred out of the ICU. Over the next several days, the patient had stable vitals and clinically improved. On hospital day 8, the patient was discharged with a NPWT device on clopidogrel 75 mg daily per the recommendation of vascular surgery. Enoxaparin sodium and warfarin were held. In total, the patient received 4 units of PRBC, 2 units of FFP, and 1 unit of platelets while hospitalized. At discharge, the patient's hemoglobin was 8.6 g/dl and platelets were 95,000/*μ*l. Warfarin 5 mg daily was resumed 20 days after discharge with an INR goal of 2.0–3.0. The wound was 5 cm long and 3 cm deep, there was minimal slough, and some granulation tissue. The patient's abdomen still had ecchymosis and there was a bulging fluid-filled area that measured 10 cm lateral to the NPWT device. The patient presented to the wound center 26 days after discharge. The wound at that time was 2.2 cm long and 2 cm deep and had healed at the skin level (Figure 3). Moderate serosanguineous drainage was present, but it was significantly less than when NPWT was initiated. Slough was minimal. NPWT was discontinued and a culture was taken. The patient was instructed to clean and pack the wound daily with iodoform packing strips and cover with a border dressing. Twenty-eight days after discharge, culture results showed rare methicillin-susceptible *Staphylococcus aureus* sensitive to doxycycline and clindamycin. Thirty-three days after discharge, a pocket was felt underneath the surface of the wound. Slight pressure was applied, and 10 ml of thick blood was drained. After draining the pocket, the wound measured 1.6 cm long and 3 cm deep. A 5 cm tunnel was also present. Clinically, there were no signs of infection, but clindamycin 300 mg three times per day for seven days was started prophylactically due to the patient's high-risk status. Clindamycin was selected because of allergy to other susceptible antibiotics including doxycycline. Dressing changes were continued as before. Forty days after discharge, the antibiotic course was complete. The wound had decreased in size, measuring 1.4 cm long and 2.4 cm deep. Tunneling had resolved. Moderate serosanguineous drainage was present. There were no signs of infection. Forty-six days after discharge, warfarin was titrated to 15 mg daily based on INR goal. Fifty-four days after discharge, the wound measured 1.2 cm long and 0.5 cm deep (Figure 4). Drainage was minimal. Surrounding tissue was soft and pliable. There were no signs of infection. The patient was instructed to discontinue wound packing and apply bacitracin to the wound site for one week and cover with a bandage.

## Discussion

Enoxaparin sodium is a widely used antithrombotic and anticoagulant agent used to prevent blood clots in patients who are high risk (1, 2). It has a rapid onset of action and for that reason is often the drug of choice if quick, short-term anticoagulation is needed. It can also be used in combination with other anticoagulants like warfarin for a predetermined amount of time to decrease the risk of blood clots during the window of time before warfarin takes effect (3, 4).

Herein, we presented a case of a patient who had significant past medical history of thrombosis and was taking clopidogrel, enoxaparin sodium, and warfarin to reinitiate anticoagulation following a recent hospitalization. Six days after beginning the anticoagulant regimen, the patient developed an expanding hematoma and hemorrhagic shock after accidentally self-administering 1 syringe of enoxaparin sodium 100 mg/ml into the left superficial epigastric artery.

This case was complicated by failure to control the patient's bleeding in a timely fashion. Liver cirrhosis contributed to low platelet count and impaired coagulation. In addition, protamine sulfate was never administered, so the effect of enoxaparin sodium was not medically reversed. The decision was made not to administer protamine sulfate due to the severity of the patient's other systemic diseases and concern about worsening underlying coagulopathy. Overdose of protamine has been shown to adversely affect clot formation and platelet function and lead to bleeding, particularly in patients with underlying coagulopathy (9, 10). Other adverse effects of protamine sulfate include anaphylaxis, rapid hypotension, and increased pulmonary artery pressures (10). Risk of these reactions would have been increased in this patient due to history of protamine exposure from insulin-controlled diabetes (10).

In addition to protamine sulfate, prothrombin complex concentrate (PCC) could have been used to reverse anticoagulation in this patient. PCC is available in two forms: 3-factor (which contains coagulation factors II, IX, and X) and 4-factor which also contains factor VI I (11). It reverses anticoagulation caused by warfarin by restoring the body's supply of vitamin K-dependent clotting factors (11). Instead of PCC, the patient in this case was given vitamin K, which also reverses warfarin but has a delayed onset of effect because its mechanism of action requires the synthesis of new clotting factors (11). PCC is a more rapid reversal technique because it immediately replenishes clotting factors. This might have been useful for this patient because, in addition to warfarin-related depletion, she likely had a baseline coagulation factor deficiency caused by liver cirrhosis and decreased synthetic function. However, she also had thrombotic history that could have made administration of PCCs risky (11).

Literature review did not reveal other cases of accidental intra-arterial injection of enoxaparin sodium, although accidental intra-arterial injection of other drugs has been reported. In general, intra-arterial injection is rare but can be associated with significant morbidity depending on what drug is injected. Drugs like benzodiazepines, penicillin, clindamycin, thiopental, phenytoin, and diclofenac are particularly high-risk if given intra-arterially (12). The exact mechanism of tissue damage is unclear but likely involves thrombosis and vasospasm leading to ischemia and necrosis (12). Development of gangrene necessitating amputation has been reported (13). Treatment depends on the offending agent, but often includes a combination of analgesics, vasodilators, steroids, anticoagulants, and thrombolytics (12). This case is unique because the offending agent was an anticoagulant, so effects like thrombosis, vasospasm, ischemia, and necrosis were not observed. The anticoagulant nature of enoxaparin sodium led to the formation of an expanding hematoma. Treatment with further anticoagulants and thrombolytics would have been inappropriate. Therefore, the main goals of treatment were to reverse anticoagulation, stop the bleeding (*via* coil embolization of the affected arteries), treat hemorrhagic shock, drain the hematoma, and then care for the resultant wound.

This case is also unique because of the location of the hematoma. Accidental injection into the superior epigastric artery is rare. Most cases of unintentional intra-arterial injection occur in the antecubital fossa where branches of the ulnar and brachial arteries run close to the surface of the skin and are easily entered (13). In addition, anomalies of these arteries are common (13).

Before evacuation, the hematoma measured 30 cm × 10 cm and contained 500 ml of blood. Draining the hematoma left the patient with a large wound on the left side of the abdomen. The wound was sufficiently deep and there was concern about continued drainage, so NPWT device was placed in the OR immediately following hematoma evacuation.

NPWT is a technique used to promote healing in complex and large wounds. It uses open-pore foam sponges, adhesive dressing, tubing, and a vacuum source to apply uniform negative pressure to all tissues on the surface of the wound (14, 15). Wound healing occurs through many mechanisms. Angiogenesis and perfusion of the wound bed are increased, increasing oxygen and nutrient delivery and waste removal (15, 16). Pro-inflammatory cytokines and proteases are removed from the interstitial fluid, reducing degradation of extracellular matrix (16). Periwound edema is reduced as fluid is pulled out (15, 16). Cell proliferation and wound contracture are mechanically stimulated by the force of the vacuum on the wound bed (16). Compared to wet or dry dressing changes, NPWT results in enhanced granulation tissue formation, decreased drainage time, greater reduction in wound bed volume, and faster wound healing (16, 17). It also decreases the infection rate for hematomas by half (16).

In this case, NPWT was discontinued 26 days after discharge, at which point the wound was reduced to 2 cm deep, drainage was significantly less than when NPWT was initiated, and granulation tissue covered the entire wound bed. The wound bed looked healthy and there were no signs of infection. A culture was taken to confirm. The wound was packed with iodoform strips and covered with a border dressing. The packing and dressing were changed daily. The patient was seen in the wound center weekly. Thirty-three days after discharge, a pocket of coagulated blood was felt under the surface of the wound. The pocket likely formed from NPWT approximating nonadjacent pieces of tissue, causing the tissue in-between to pinch off, and the resultant potential space filled with blood. The pocket was drained, and prophylactic antibiotics were given based on culture results and patient allergies. The remainder of wound healing was uncomplicated. The wound and pocket were packed with iodoform strips and covered with a border dressing. The packing and dressing were changed daily. The wound healed by secondary intention.

## Limitations

The limitations of this article include its design as a single case. Additionally, there are no other reports of accidental intra-arterial injection of enoxaparin sodium available, so discussion of the general risks and management principles of accidental intra-arterial injection are based on case reports involving intra-arterial injection of other drugs such as benzodiazepines, penicillin, clindamycin, thiopental, phenytoin, and diclofenac.

## Conclusion

This report describes an unusual case of accidental intra-arterial injection of enoxaparin sodium with expanding hematoma formation in a patient with significant thrombotic history who was completing an enoxaparin sodium bridge to warfarin. Clinicians should be vigilant of early signs of expanding hematoma formation and, if present, stop anticoagulant treatment, transfuse, and administer vasoconstrictors as necessary to minimize bleeding and prevent the development or worsening of hemorrhagic shock. Arterial coil embolization and surgical evacuation of the hematoma may also be necessary. After the hematoma is evacuated, the resultant wound can be treated with NPWT until wound depth is sufficiently reduced and drainage is minimal, at which time the wound can be allowed to heal by secondary intention with regular dressing changes while monitoring for potential complications. When caring for a wound that was previously treated with NPWT, be cognizant that a blood-filled pocket can form underneath the surface of the wound if non-adjacent pieces of tissue are approximated. If this occurs, the pocket must be drained as it is a potential nidus of infection.

## Data Availability

The original contributions presented in the study are included in the article/Supplementary Material, further inquiries can be directed to the corresponding authors.
